# Evolution of Glaucoma Surgery in the Last 25 Years

**DOI:** 10.5041/RMMJ.10345

**Published:** 2018-07-30

**Authors:** Laura Bar-David, Eytan Z. Blumenthal

**Affiliations:** 1 Department of Ophthalmology, Rambam Health Care Campus, Haifa, Israel; 2 The Ruth & Bruce Rappaport Faculty of Medicine, Technion–Israel Institute of Technology, Haifa, Israel

**Keywords:** *Ab interno* glaucoma surgery, glaucoma, micro-stents, minimally invasive glaucoma surgery

## Abstract

Glaucoma is a chronic neurodegenerative optic nerve disease. Treatment is intended to prevent the development and progression of optic nerve damage by lowering intraocular pressure (IOP). Current therapy options include topical/systemic drugs that increase aqueous humor outflow or decrease its production, laser therapy that targets the trabecular meshwork and ciliary body, and incisional surgery. Trabeculectomy as well as glaucoma drainage devices are often performed, given their high efficacy in lowering IOP. However, the significant risk profile with potential sight-threatening complications has motivated glaucoma experts to create alternative surgeries to treat glaucoma. Minimally invasive glaucoma surgery (MIGS) is defined by: micro-invasive approach, minimal tissue trauma, high safety profile, and rapid recovery. The new devices might promote an earlier transition from medical/laser therapy to surgery, and therefore decrease the side effects associated with long-term use of topical medications as well as deal with the limited adherence of patients to their regimens. This review presents the surgical options available for glaucoma patients and their evolution over the past 25 years.

## INTRODUCTION

Glaucoma is the second leading cause of blindness worldwide,^1^ and its chronic nature requires treatment throughout the patient’s lifetime. Intraocular pressure (IOP) is currently the only known modifiable risk factor,^2^ thus nearly all existing therapeutic modalities have a common goal of reducing IOP via medications, laser, or surgery. Surgery is typically performed when non-invasive efforts (maximal tolerated medical therapy and/or laser trabeculoplasty) have been exhausted and are incapable of reaching target IOP levels (IOP levels preventing further visual field damage). In terms of incisional surgery for glaucoma, trabeculectomy is considered the gold standard, with tube shunt procedures trailing shortly thereafter.^3^ Both trabeculectomy and tubes are well established procedures that have been successfully employed for several decades.^4^ Although highly efficacious, they are associated with intense postoperative care for the first 2–3 months and carry the risk of potential vision-threatening complications.^5^ More recently, less invasive glaucoma procedures, collectively termed MIGS (minimally invasive glaucoma surgery), have gained popularity, with new devices entering the market regularly. Though the criteria for meeting the definition of a MIGS procedure is somewhat controversial, they all share common characteristics of IOP reduction with reduced tissue destruction, a relatively high safety profile, short surgery time, simple instrumentation, and rapid recovery.^6^ Due to their high safety profile, MIGS may be considered in milder diseases as opposed to more invasive procedures. Thus, for patients with milder diseases, MIGS potentially broaden the therapeutic options that were usually only non-invasive, while traditional surgeries will keep targeting patients with more advanced disease.^7^ There are three main aqueous outflow pathways which have been the center of attention for MIGS devices: Schlemm’s canal improving trabecular outflow, the suprachoroidal space improving the uveoscleral outflow, and the subconjunctival space creating an alternative outflow pathway. Most studies compare the efficacy of these procedures based on the following three parameters: visual acuity, IOP, and number of topical therapies used. The purpose of this review is to summarize surgical procedures available for glaucoma patients, their indication, efficacy, safety, cost-effectiveness, and how they fit together in the overall management of glaucoma patients (Table 1).

**Table 1:** Comparison of Glaucoma Surgical Procedures

Outflow Pathway	Surgery	IOP Reduction	Medication Reduction
Subconjunctival	Trabeculectomy^4^	49.5% at 5 years	1.5 at 5 years
	Aqueous shunt^4^	41.4% at 5 years	1.4 at 5 years
	Ex-PRESS^8^	44% at 2 years	3.4 at 2 years
	XEN^9^	36.4% at 1 year	1.8 at 1 year
Trabecular	iStent^10^	20% at 1 year	1.2 at 1 year
	Hydrus*^11^	36% at 2 years	1.5 at 2 years
	Trabectome^12^	52% at 1 year	1.3 at 1 year
Suprachoroidal	Cypass*^13^	30% at 2 years	1.2 at 2 years

* Combined with cataract surgery

## METHODS

An online PubMed search for key words including glaucoma surgery, minimally invasive glaucoma surgery, and the names of specific procedures was performed.

### Traditional Glaucoma Surgeries

#### Trabeculectomy

Trabeculectomy remains the gold standard surgical procedure for achieving target IOP in glaucoma patients.^3^ During this procedure, a “fistula” is created between the anterior chamber and the sub-tenon space in order to allow the aqueous humor to bypass the trabecular meshwork. An opening (ostomy) is performed in the corneoscleral tissue situated at the level of the trabecular meshwork. This ostomy is covered by a scleral flap, converting it to a “guarded filtration” (in contrast to “full-thickness” procedures that are no longer performed), providing relative control of the outflow rate, although the tightness of the flap sutures results in variable resistance and hence lack of standardization. The aqueous humor drains under the scleral flap to a sub-tenon (occasionally referred to as subconjunctival) reservoir called a “bleb,” hidden behind the upper eyelid. During the procedure a peripheral iridectomy is created, to avoid the obstruction of the internal opening of this newly created ostomy by the iris.^14^

One of the most important challenges of a successful trabeculectomy surgery is to prevent scarring of the sub-tenon space which would limit, and ultimately block, the outflow of aqueous humor from the anterior chamber. Postoperative fibrosis of the filtration path, which usually occurs during the first few weeks to months after surgery, is the main cause of postoperative failure. Hence, antimetabolites aimed at preventing tissue scarring are routinely employed during surgery in order to reduce the likelihood of trabeculectomy failure.^15^ The first antimetabolite used during trabeculectomy surgery was 5-fluorouracil (5-FU); however, a survey by the American Glaucoma Society in 2008 reported that 85%–99% of surgeons preferred mitomycin-C (MMC) over 5-FU.^16^ In order to locally apply the antimetabolite at a high concentration at the needed surgical site, a surgical sponge is soaked with the antimetabolite at various concentrations and then placed on the sclera (for variable amounts of time) where the surgeon plans to perform the ostomy. Patients with underlying characteristics and risk factors for trabeculectomy failure often require higher concentrations and longer durations of antimetabolite exposure. Thereafter, a critical step to ensure safety is meticulous irrigation of the ocular surface in order to remove the antimetabolite. Alternatively, antimetabolites may be injected preoperatively into the sub-tenon space in predefined volume and concentration, titrating their long-lasting antifibrotic effect. Postoperative care and surveillance is essential for the success of trabeculectomy surgery. Early postoperative complications include leakage, choroidal effusion, hypotony, a shallow anterior chamber, and hyphema. Early hypotony, a dreaded complication, may lead to vision loss in up to 20% of patients.^17^ Long-term complications include leakage, failure, bleb infection, endophthalmitis, and long-term ocular surface irritation.^5^ Management of a failing or failed trabeculectomy includes suturelysis which is a tight laser beam aimed at the scleral suture, needling aimed at breaking fibrotic adhesions,^18^ an additional trabeculectomy procedure, or a glaucoma drainage device procedure. A repeat trabeculectomy is associated with a higher complication rate and an increased risk of subsequent failure.

#### Tube Shunt Surgery

Aqueous shunts, also called glaucoma drainage devices, are artificial filtering devices that lower the IOP by draining aqueous humor via a tube into the subconjunctival space. These silicone tubes drain aqueous humor from the anterior chamber to a plate of variable size and shape placed on the sclera, usually in the superotemporal quadrant.^19^ They initially were indicated in cases where trabeculectomy was likely to fail, such as in neovascular glaucoma, iridocorneal endothelial syndrome, aphakic glaucoma, Sturge–Weber syndrome, glaucoma after vitreoretinal surgery or keratoplasty, and uveitic glaucoma.^20^ With time, the use of tube shunt devices in glaucoma management has become increasingly popular, even as a first procedure, after its favorable success rate has been shown in a number of clinical studies.^21^ The Tube Versus Trabeculectomy (TVT) study (see later) is a pivotal trial that compared tube shunt surgery to trabeculectomy.^22^

Aqueous shunts differ in material, plate shape, size, thickness, and the presence (or absence) of a valve. The Molteno implant was the first commercially available tube shunt; currently, the Ahmed glaucoma valve (AGV) (New World Medical Inc., Rancho Cucamonga, CA, USA) and Baerveldt glaucoma implant (BGI) (Abbott Medical Optics, Abbott Park, IL, USA) are the most commonly used.^23^

Intraoperative complications are infrequent, the most common being hyphema during tube insertion. Hypotony, a shallow anterior chamber, tube-cornea touch, corneal edema, uncontrolled high pressure, ptosis, and diplopia may occur in the early postoperative period. While early complications may be related to surgical technique, late complications are less predictable. They include corneal edema, erosion, persistent motility disturbance, chronic iritis, tube obstruction, failure of intraocular pressure control, and rarely endophthalmitis. Endophthalmitis following aqueous shunts is rare and far less common than after trabeculectomy. The single risk factor for endophthalmitis is tube exposure, and if this is present it should be revised urgently.^24^

#### The Tube Versus Trabeculectomy Study

Trabeculectomy has been the procedure of choice with tube surgery reserved for cases at high risk for trabeculectomy failure (repeated trabeculectomy failure, neovascular glaucoma, uveitic glaucoma, and others).^20^ Due to the risk of bleb-related complications, tube procedures are often considered as a possible alternative.^21^ The purpose of the Tube Versus Trabeculectomy (TVT) study was to compare the safety and efficacy of tube procedures versus trabeculectomy with MMC in patients with uncontrolled glaucoma who had previously undergone cataract surgery and/or failed trabeculectomy.^22^

The TVT study, surprisingly, did not demonstrate clear superiority of one glaucoma operation over the other, but indicated that both tube shunt surgery and trabeculectomy with MMC are viable surgical options for treating medically uncontrolled glaucoma in patients with previous cataract extraction and/or failed filtering surgery.^4^ Both tube shunt and trabeculectomy with MMC were effective in lowering IOP, with a 41.4% and 49.5% decrease, respectively, at 5-year follow-up. Based on the use of supplemental glaucoma medication therapy, the overall success rate was higher for the tube group after 5 years. The trabeculectomy group had a progressive increase in adjunctive medical therapy during 5 years of follow-up, while the use of glaucoma medications remained relatively constant in the tube group. Rates of failure of trabeculectomy and tube shunt were similar and average approximately 10% per year; inadequate IOP reduction was the most common reason for failure in both treatment groups. Failure from persistent hypotony happened more frequently in the trabeculectomy group. The rate of reoperation for glaucoma was higher in the trabeculectomy group, but additional glaucoma surgery in the tube group happened to be more complex and eventually involved placement of a second tube shunt or cyclodestruction. Reduction in visual acuity occurred similarly in both treatment groups during 5 years of follow-up. The TVT study presented similar rates of long-term complications, as well as success, in the BGI and trabeculectomy groups at 5 years.

Even though randomized clinical trials like the TVT study offer a high level of evidence-based medicine, we must consider other factors when choosing a glaucoma surgery. The risk profile of the patient’s eye, the surgeon’s skills and experience with each surgical option, the patient’s willingness to undergo repeat glaucoma surgery, and the surgeon’s understanding of the case are all important factors in the surgery decision-making process. The benefits of tube shunt surgery versus trabeculectomy with MMC in reducing IOP must be interpreted in the context of their potential adverse events on one hand, and their potential beneficial effects on the other.

### Minimally Invasive Glaucoma Surgery

#### Trabecular Meshwork Bypass

##### iStent (Glaukos Corporation, Laguna Hills, CA, USA)

The iStent is a 1-mm-long and 0.3-mm-wide trabecular micro-bypass, made from heparin-coated titanium. It is the smallest Food and Drug Administration (FDA)-approved medical device to be implanted in human beings. It comes within a preloaded injector and is inserted *ab interno* through the trabecular meshwork into Schlemm’s canal under direct gonioscopic view,^25^ and, bypassing the juxtacanalicular trabecular meshwork (the site of major resistance^26^), the iStent enables enhanced aqueous humor flow, resulting in lower IOP. Since initial development of the iStent 15 years ago, several publications report its efficacy as an isolated procedure or in combination with cataract surgery. The iStent study group conducted in 2011 a large randomized controlled trial comparing the results of cataract surgery alone versus combined with a single iStent injection.^27^ The reduction in the IOP was minor, statistically significant, in patients who received the combination surgery compared to those who underwent cataract surgery alone: 72% of patients who received the combination surgery kept IOP ≤21 mmHg after 12 as well as 61% after 24 months, whereas only 50% of the cataract-only group did so at both time points. A decrease in the amount of glaucoma medication was also achieved in both groups (1.4±0.8 in the combination surgery group as opposed to 1.0±0.8 in the cataract group). These results support the claim that reduction in IOP secondary to a combined iStent implantation–cataract surgery outweighs the effect of cataract surgery alone.^10^ Other studies demonstrated that this IOP reduction difference persists long-term.^28^ Data collected during such clinical trials suggested better results with multiple versus a single iStent implantation,^29^ hence, Glaukos developed a second-generation device called iStent Inject, designed for implantation of two stents in a single surgical procedure.^30^ A prospective study evaluated the efficacy of two iStent Inject devices compared to two antiglaucoma medications in patients with mild to moderate open angle glaucoma. After 1 year, 94.7% of the iStent Inject group and 91.8% of the antiglaucoma medications group had a ≥20% reduction in IOP, demonstrating that the insertion of two iStent Inject devices had comparable efficacy to two topical medications.^31^ These results suggest that the iStent Inject may be considered an alternative to topical medication as first-line therapy. No studies have yet been published on the effect of iStent on more advanced and progressive disease.

##### Hydrus microstent (Invantis Inc., Irvine, CA, USA)

The Hydrus microstent is a biocompatible nitinol (an alloy of nickel and titanium) 8-mm-long trabecular bypass device. It is an intracanalicular scaffold designed to stent and dilate Schlemm’s canal allowing increased outflow through its three openings. Implantation is also here performed via the *ab interno* route, such that the preloaded injector is passed through a corneal incision opposite the implantation site. A randomized clinical trial comparing cataract surgery alone versus a combined Hydrus and cataract surgery demonstrated that 80% of the patients from the combined surgery group had 20% decrease in IOP at 24 months, versus 46% in the cataract surgery group.^11^ Another study compared the efficacy of the Hydrus to selective laser trabeculoplasty (SLT) patients with glaucoma.^32^ Both groups had significant decrease in IOP after 12 months: in the Hydrus surgery group 47% of the patients were medication-free at 12 months, compared to only 4% of the patients in the SLT group.

##### Trabectome (Neomedix Corporation, Tustin, CA, USA)

In the Trabectome procedure an electrode performs thermal ablation of both the trabecular meshwork and the canal of Schlemm’s inner wall. The *ab interno* approach, which is done under direct gonioscopic view, creates a bypass of the trabecular meshwork resistance to outflow of aqueous fluid by creating a straight communication between the anterior chamber and the lumen of the canal of Schlemm, which thereafter communicates with the collector channels. The Trabectome Surgeon Database is a large data set evaluating the efficacy of the Trabectome procedure. Most conducted trials have compared the Trabectome alone versus a combined Trabectome with cataract surgery.^12^,^33^ Overall, a statistically significant reduction was found in both IOP and amount of antiglaucoma medications in both groups. Reported complications included most commonly a minor, and expected, blood reflux from the canal of Schlemm with a resulting self-limited hyphema (blood pooled at the anterior chamber).

#### Suprachoroidal Space Implants: Cypass Micro-Stent (Transcend Medical, Menlo Park, CA, USA)

The Cypass is a flexible fenestrated polyamide 6.4-mm-long tube inserted under gonioscopic view, *ab interno*, placed within the potential space among the sclera and the ciliary body. It enhances aqueous outflow by providing a straight communication between the anterior chamber and the suprachoroidal space. The COMPASS study is the largest trial investigating the efficacy of the Cypass. Patients were randomized to either the Cypass combined with cataract surgery or cataract surgery alone. After 2 years, 72.5% of cases that were treated with a combined procedure obtained a reduction in 20% in IOP, versus 58% in the cataract alone group. The investigators further reported that 61.5% of eyes treated with the combination surgery maintained IOP between 6 and 18 mmHg without any medication, versus 43.5% for those that underwent cataract alone.^13^

#### Subconjunctival Space Implants

Devices targeting the subconjunctival space use the same traditional approach as more conventional glaucoma surgeries of creating a new outflow pathway leading to the subconjunctival space.^34^ Unlike the traditional filtration surgery options, trabeculectomy and tubes, these newer MIGS approaches do not require conjunctival incision and have a fixed predefined lumen diameter restricting flow to decrease the hypotony risk.

##### Ex-PRESS miniature glaucoma shunt (Alcon Laboratories Inc., Fort Worth, TX, USA)

The Ex-PRESS miniature glaucoma implant is a biocompatible stainless-steel tube intended to drain the aqueous fluid into the subconjunctival space. For implantation a scleral flap needs to be created as well as a filtration bleb in the conjunctiva, similarly to standard trabeculectomy, but without the need for a peripheral iridectomy.^35^ The Ex-PRESS shunt gained popularity because its use is similar to standard trabeculectomy, but with a somewhat less invasive procedure.^36^ The standardized lumen diameter and length translate to a fixed predefined resistance to flow, providing more uniform filtration. Complications and rates of failure are similar to the ones reported in the TVT study.^37^ Theoretically, the Ex-PRESS device offers a steadier flow of aqueous humor compared to the trabeculectomy thanks to the standardized size of the lumen, and the bleb is thus small and diffuse. It has been argued that the Ex-PRESS device can also be helpful in patients with scarring since it might simply need a smaller quantity of intact conjunctiva for its placement.

Some studies compare the Ex-PRESS miniature glaucoma shunt and the trabeculectomy. Most of these studies described that there is no significant difference in the decrease of IOP when comparing the two procedures.^38^,^39^ The single advantage distinguished with the Ex-PRESS shunt was a decreased rate of postoperative hypotony in early stages, while most studies report no difference in the long-term follow-up.^40^,^41^ Ex-PRESS success rates were not found to correlate with patient characteristics or with eyes that underwent prior glaucoma surgery, such that this procedure might be relevant to an extensive variety of glaucoma types.

One study reported that the cost for Ex-PRESS surgery is 3.5 times higher when than trabeculectomy ($1,203 and $339, respectively). This study concluded that trabeculectomy is significantly more cost-effective for IOP reduction in patients with glaucoma.^42^

##### XEN gel implant (Allergan, AqueSys Inc., Aliso Viejo, CA, USA)

The XEN gel implant is a 6-mm-long soft cross-linked porcine collagen implant. Inserted *ab interno* from a preloaded injector, it creates a drainage communication between the anterior chamber and the subconjunctival space.^43^ Unlike other MIGS procedures, it does not require gonioscopic view during the surgery. The 45-nm lumen version is the only model currently available, because larger lumen implants produced early postoperative hypotony.^9^

A recent study of XEN implantation combined with phacoemulsification showed IOP reduction to ≤18 mmHg without medications in 90% of the patients at 1 year. Regarding the safety profile of the XEN implantation procedure, the complications reported were intraoperative as well as postoperative, were less severe, and related to the surgical procedure, for instance subconjunctival bleeding and anterior chamber hyphema during the implantation; these complications were self-limited.^43^ Several studies compared the efficacy of the XEN gel implant to the traditional trabeculectomy. A retrospective interventional cohort study evaluated the risk of surgical failure in patients who underwent XEN gel implantation compared to trabeculectomy. The IOP decrease between the two groups had non-statistically significant differences. Failure was described as IOP <6 mmHg in addition to vision loss or an IOP >17 mmHg without medications on two consecutive follow-ups, a need for another operation, or visual acuity of no light perception. The failure rate was non-statistically significant between the two procedures. Of the groups mentioned, 25% of XEN gel implant cases and 33% of trabeculectomy cases received antiglaucoma medications at the last appointment. The most common additional procedure needed was needling, which was performed more often in the XEN gel implant group.^44^,^45^ All studies reported good efficacy and a favorable safety profile after 1-year follow-up.

## DISCUSSION

Considering that glaucoma is a neurodegenerative chronic disease, the goal of glaucoma treatment and surgery is preserving the quality of life (QOL) of the patients, and their independence.^46^ The therapeutic tactic is meant to maintain visual acuity as well as visual field in addition to reducing interference of the disease with daily life, including decreasing antiglaucoma therapy and its potential ocular and systemic adverse effects. The large number of new glaucoma devices commercialized this past decade reflects the inability of the time-honored options to deliver the goal with a reasonable risk profile. Using 12 quality of life parameters established to evaluate glaucoma patients, it was shown that the quality of life can be maintained with trabeculectomy, iStent, and Trabectome procedures.^47^,^48^ Social functioning, one of the categories of the QOL index, shows a meaningful increase with MIGS. The IOP and the number of glaucoma medications used are all considered to be major QOL determinants in glaucoma, given their influence on everyday activity, although not yet part of the QOL questionnaire. Although the authors could not identify a difference in the QOL score between trabeculectomy and MIGS, the trabeculectomy cohort confirmed a more remarkable reduction in IOP and glaucoma medication than the MIGS cohort at various points in time.

The efficacy of the majority of MIGS procedures in lowering IOP is lower than the more invasive glaucoma procedures, trabeculectomy with MMC and tubes. This compromise in efficacy is compensated by a much higher safety profile, as the traditional surgeries are associated with potential sight-threatening complications. A broad range of intraoperative and postoperative complications associated with MIGS have been reported, most of which are transient and resolve with time and with medical management. In the early postoperative course micro-stent obstruction can develop and may require additional surgery. Hypotony and its consequences are relatively infrequent given that most MIGS have been specifically designed to avoid very low IOPs, such that most occurrences of early hypotony resolve with conservative management.^49^,^50^

Most of the studies comparing the efficacy of MIGS to cataract surgery show that MIGS is more effective in reducing IOP when compared to cataract surgery alone, and given that MIGS can be easily performed in combination with cataract surgery the effect may be more substantial.^51^,^52^ Still, the gold-standard method against which new surgical procedures for reducing IOP in glaucoma are compared is trabeculectomy, due to its high efficacy and success rates also in cases of more advanced glaucoma. In contrast with these more advanced, complicated, and high-risk cases, most MIGS clinical trials have restricted themselves to evaluating and comparing procedures in cases of mild to moderate stages of glaucoma. Therefore, data on MIGS, to date, do not typically exist for cases of more severe disease. With the further accumulation of data and knowledge on MIGS, appropriate patient selection for each procedure, as well as more precise indications, should allow us to tailor specific procedures to the individual needs of each eye harboring uncontrolled glaucoma. For example, MIGS targeting trabecular outflow could benefit mild to moderate glaucoma because the reduction in IOP is limited by the episcleral venous pressure.^53^ Minimally invasive glaucoma surgery creating subconjunctival drainage target the cornerstone of glaucoma surgery and may achieve lower IOP, but with less frequent bleb-related complications and hypotony. Minimally invasive glaucoma surgeries enhancing the suprachoroidal outflow share the advantage of not being limited by the episcleral venous pressure, and, in addition, do not share the risks of a bleb-forming procedure. Therefore, it is probable that these new MIGS procedures, instead of replacing traditional surgeries, will each find its niche depending on the risk/benefit profile desired in each individual case. Because of the improvement in efficacy and risk profile, a change should take place toward higher utilization of MIGS in the algorithm for the treatment of glaucoma. In mild to moderate glaucoma, surgery may be proposed earlier and reduce the need for topical medication and its ocular surface toxicity, lifetime cost, and often limited compliance. This role will be clarified as more information becomes available, especially well-designed prospective randomized clinical trials comparing invasive surgical procedures against each other, with or without cataract surgery. Besides the information on the effectiveness of MIGS, limited evidence is available on the cost-effectiveness of MIGS—whether the cost of the device is outweighed by cost savings from decreased medication and need for further interventions or management of complications. That is why these studies should best focus not only on IOP and medication but also report issues related to cost-effectiveness and QOL, in early, moderate, as well as far-advanced disease.

## Abbreviations

5-FU: 5-fluorouracil
AVG: Ahmed valve glaucoma
BGI: Baerveldt glaucoma implant
IOP: intraocular pressure
MIGS: minimally invasive glaucoma surgery
MMC: mitomycin-C
QOL: quality of life
SLT: selective laser trabeculoplasty
TVT: tube versus trabeculectomy.
